# Gene expression profiles help identify the Tissue of Origin for metastatic brain cancers

**DOI:** 10.1186/1746-1596-5-26

**Published:** 2010-04-26

**Authors:** Alan HB Wu, Julia C Drees, Hangpin Wang, Scott R VandenBerg, Anita Lal, William D Henner, Raji Pillai

**Affiliations:** 1 Department of Laboratory Medicine, University of California, San Francisco, CA-94143, USA; 2 Pathwork Diagnostics, Redwood City, CA-94063, USA; 3 Department of Pathology, Division of Neuropathology, University of California, San Diego, CA-92093, USA; 4 Guangzhou First Municipal People's Hospital and Guangzhou Medical College, China

## Abstract

**Background:**

Metastatic brain cancers are the most common intracranial tumor and occur in about 15% of all cancer patients. In up to 10% of these patients, the primary tumor tissue remains unknown, even after a time consuming and costly workup. The Pathwork^® ^Tissue of Origin Test (Pathwork Diagnostics, Redwood City, CA, USA) is a gene expression test to aid in the diagnosis of metastatic, poorly differentiated and undifferentiated tumors. It measures the expression pattern of 1,550 genes in these tumors and compares it to the expression pattern of a panel of 15 known tumor types. The purpose of this study was to evaluate the performance of the Tissue of Origin Test in the diagnosis of primary sites for metastatic brain cancer patients.

**Methods:**

Fifteen fresh-frozen metastatic brain tumor specimens of known origins met specimen requirements. These specimens were entered into the study and processed using the Tissue of Origin Test. Results were compared to the known primary site and the agreement between the two results was assessed.

**Results:**

Fourteen of the fifteen specimens produced microarray data files that passed all quality metrics. One originated from a tissue type that was off-panel. Among the remaining 13 cases, the Tissue of Origin Test accurately predicted the available diagnosis in 12/13 (92.3%) cases.

**Discussion:**

This study demonstrates the accuracy of the Tissue of Origin Test when applied to predict the tissue of origin of metastatic brain tumors. This test could be a very useful tool for pathologists as they classify metastatic brain cancers.

## Background

Metastatic tumors to the brain are the most common central nervous system (CNS) neoplasm and occur in about 15% of all cancer patients. In an adult, these tumors originate most frequently from lung, breast, skin, kidney and colon [1]. In up to 10% of patients, no clear primary site can be determined despite an intensive clinical evaluation [2,3]. Current treatment regimens for these patients with unknown primaries include surgery, radiation and chemotherapy [4,5]. Despite these treatments, the survival rates of patients with brain metastases remain low. A recent retrospective review of 740 patients with brain metastases of all tissue types reported actuarial survival rates of 8.1% at 2 years and 2.4% at 5 years [6].

The last decade has seen the emergence of a number of markers of immunohistochemistry (IHC) that have been helpful in indicating the origin of common metastatic tumors to the brain [7,8]. Workup of an immunohistochemical signature requires selection and use of a large number of antibodies and knowledge of their reactivities (and cross-reactivities) to different cancer types. A recently proposed algorithm for immunohistochemical evaluation of the common types of brain metastases recommends the use of 18 antibodies to distinguish 10 cancer types [7]. Immunohistochemical markers with a high degree of sensitivity and that are also highly specific for a single primary site are uncommon. In addition, interpretation of immunohistochemical stains is user-dependent and requires subjective interpretation. Even after a rigorous workup, success rates at identifying primary tumor sites are not optimal [9]. A meta-analysis of four studies in which pathologists were blinded to the knowledge of the primary site showed that even an extensive IHC workup correctly identified the primary site for only 66% of all metastatic specimens [9]. Additional diagnostic approaches are required to complement more traditional IHC analysis.

Diagnostic molecular profiling assays that use either microarrays or real-time reverse transcription polymerase chain reaction (RT-PCR) have been developed to identify the tissue of origin of metastatic cancers [10,11]. Among these, microarray-based assays have the advantage of simultaneously evaluating the gene expression pattern of thousands of genes. The information generated is mined using multigene classifiers that predict the tissue of origin. The Pathwork^® ^Tissue of Origin Test (Pathwork Diagnostics, Redwood City, CA, USA) for frozen specimens uses microarray technology to measure the gene expression pattern, comprising 1550 genes, of a tumor with an uncertain origin and compares it to expression patterns of a panel of 15 known tumor types. The tissue types represented are: bladder, breast, colorectal, gastric, hepatocellular, kidney, non-small cell lung, ovarian, pancreatic, prostate, thyroid, melanoma, testicular germ cell, non-Hodgkin's lymphoma, and sarcoma. This panel represents approximately 90 percent of all solid tumors and 58 morphologies overall. In validation studies, the Tissue of Origin Test had an accuracy of 87.8% in a set of 547 frozen specimens and delivered reproducible results (93.8% concordance) in four different laboratory settings [10,12].

The purpose of this study was to evaluate the performance of the Pathwork Tissue of Origin Test in identifying the primary site for metastatic brain cancer patients. Fifteen cases of metastatic brain cancers of known origins were processed through the Tissue of Origin Test, and predicted the accurate tissue of origin in 92.3% of cases that met specimen entry criteria. These results show that the Tissue of Origin Test is a useful diagnostic tool to aid physicians treating metastatic brain cancer patients.

## Methods

### Tumor Specimens

Tumor specimens that met the following criteria were entered into the study: excisional biopsies of brain metastases, fresh-frozen (100 mg) samples available for analysis, sample contained at least 60% tumor and < 20% necrosis and the primary site of the tumor was known. Sixteen fresh-frozen brain metastases were obtained from the UCSF Neurological Surgery Tissue Bank using protocols approved by the UCSF Committee of Human Research. One additional frozen brain metastasis tumor specimen was obtained from Cytomyx (Rockville, MD). In addition, microarray data files for seven frozen brain metastases were obtained from Gene Logic, Inc. (Gaithersburg, MD) and one from Gene Expression Omnibus (GEO; Accession number GSM76622). When available, Hematoxylin and Eosin (H&E) sections adjacent to the tumor sample were reviewed by a pathologist to determine the percentage of tumor cells and necrosis. In all cases, the primary tissue site of the brain metastasis was known.

### RNA Extraction, Target Preparation, and Microarray Processing

Specimens were processed as described earlier [10,12]. Total RNA was extracted using the RNeasy Midi kits (Qiagen Inc., Valencia, CA) following manufacturer-recommended protocols and as described earlier [12]. Total RNA concentration was assessed by spectrophotometry (OD 260 nm), and the purity was judged by the ratio of absorbance at 260 nm to 280 nm (A_260_/A_280_). Biotin-labeled cRNA was synthesized using GeneChip expression assay reagents and protocols (Affymetrix, Inc, Santa Clara, CA), and the samples were hybridized to the Pathwork Diagnostics Pathchip using commercially available reagent kits and protocols (Affymetrix, Inc., Santa Clara, CA). The arrays were scanned using the Affymetrix GCS3000 Scanner. The resulting raw intensity data files (CEL files) were processed at Pathwork Diagnostics for automated analysis and report generation. The CEL files contain raw data on RNA expression in each tumor. Only CEL file that have greater than a minimum threshold of overall signal of 20 are used for further analysis. The Tissue of Origin Test utilizes the expression levels of 1550 genes to compute a similarity score to each of 15 cancer types as described earlier [10].

## Results

### Tumor Specimens and Quality Control

The 25 brain metastases specimens were reviewed for eligibility in the study. Fifteen specimens met all criteria and were entered into the study. Patient demographics for these 15 specimens are summarized in Table 1. In all cases, the biopsy site was brain. The most common primary sites were breast and lymphoma (3/15 cases each), followed by lung and melanoma (2/15 cases each). The most common specimen morphology was adenocarcinoma and all morphologies were consistent with the available diagnoses (Table 2). The majority (77.8%) of the brain metastases were located in the cerebral hemispheres while a smaller number of cases (22.2%) were located in the cerebellum (Table 2).

**Table 1:** Patient Demographics.

Primary Site (n = 15)	
**Tissue Type**	**No. of Cases**
Lung	2
Breast	3
Melanoma	2
Lymphoma	3
Sarcoma	1
Colon	1
Head & Neck	1
Gastric	1
Kidney	1

Patient Age Years (n = 6)*	
Median	41
Range	21-56

**Patient Gender (n = 6)**	
Male	3
Female	3
Not specified	9

* Only 6/15 cases had information on age available

**Table 2:** Clinical Characteristics of Brain Metastases Cases.

Case ID	Available Diagnosis	Specimen Morphology	Location within Brain
A	Sarcoma	Sarcoma	Temporal-Parietal
B	Kidney	Renal cell carcinoma	Temporal-Occipital
C	Melanoma	Malignant melanoma	Parietal Lobe
D	Melanoma	Malignant melanoma	Frontal Lobe
E	Breast	N/A	Cerebellum
F	Breast	Carcinoma	N/A
G	Lung	Poorly differentiated carcinoma	Frontal Lobe
H	Lymphoma	Diffuse large B-cell lymphoma	Temporal Lobe
I	Lymphoma	Diffuse large B-cell lymphoma	N/A
J	Colon	Adenocarcinoma	Temporal Lobe
K	Breast	Adenocarcinoma	Cerebellum
L	Head & Neck	Squamous Cell Carcinoma	N/A
M	Gastric	Adenocarcinoma	N/A
N	Lung	Adenocarcinoma	N/A
O	Lymphoma	Diffuse large B-cell lymphoma	N/A

### Tissue of Origin Test Results

**Figure 1:**
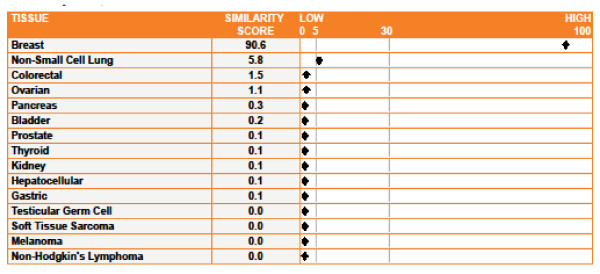
**Sample Tissue of Origin Test Report**. The Tissue of Origin Test report for specimen with case ID K in this study is shown as a sample report. The report presents similarity scores to each of the 15 tissue types included in the Tissue of Origin Test panel. A similarity score of 90.6 indicates a high confidence that this specimen is a breast cancer metastasis to the brain. Also, the Tissue of Origin Test excluded 13 tissue types (those with similarity scores less than or equal to 5) as the likely tissue origins for this specimen.

The Tissue of Origin Test produces a similarity score for each of the 15 tissue types included in the Tissue of Origin Test panel. A sample report (specimen with Case ID K in this study) is shown in Figure 1. The similarity score is a measure of the similarity of the gene expression profile of the specimen to the profile of the indicated tissue, ranging from 0 (very low similarity) to 100 (very high similarity). Similarity scores for all 15 tissues sum to 100. In addition, any tissue with a similarity score less than or equal to 5 has a 99.8% probability of not being on the tissue of origin. Thus, the Tissue of Origin Test can be used to exclude, or rule out, origins from specific tissues on the Test panel.

Fourteen of the 15 specimens entered into the study were processed successfully to yield qualified data files and Tissue of Origin Test results (Table 3, complete results in Additional File 1). The specimen that did not yield a qualified data file had low overall signal intensity. Another specimen was head and neck which is not one of the 15 tissues represented on the Tissue of Origin Test panel. For the thirteen on-panel specimens, the Tissue of Origin Test accurately predicted the primary in 92.3% of the cases. Overall, including the off-panel specimen, the Tissue of Origin Test accurately predicted the primary in 12/14 (85.7%). The one non-agreement between the Tissue of Origin Test result (ovarian carcinoma) and the clinically available diagnosis (lung carcinoma) was seen in a female that was histopathologically diagnosed as a metastatic adenocarcinoma.

**Table 3:** Tissue of Origin Test Results for Individual Brain Metastases Cases.

Case ID	Qualified Data File	Available Diagnosis	Tissue of Origin Test Result	Highest Similarity Score	Agreement with Available Diagnosis
E	Pass	Breast	Breast	90.6	Yes
F	Pass	Breast	Breast	81	Yes
K	Pass	Breast	Breast	90.5	Yes
H	Pass	Lymphoma	Lymphoma	90.4	Yes
I	Pass	Lymphoma	Lymphoma	87.7	Yes
O	Pass	Lymphoma	Lymphoma	94.4	Yes
G	Pass	Lung	Lung	62.6	Yes
N	Pass	Lung	Ovarian	89.4	No
C	Pass	Melanoma	Melanoma	77	Yes
D	Pass	Melanoma	Melanoma	75.9	Yes
J	Pass	Colon	Colon	82.4	Yes
M	Pass	Gastric	Gastric	35	Yes
A	Pass	Sarcoma	Sarcoma	65.2	Yes
L	Pass	Head & Neck	Lung	77.3	Off-Panel

**Figure 2:**
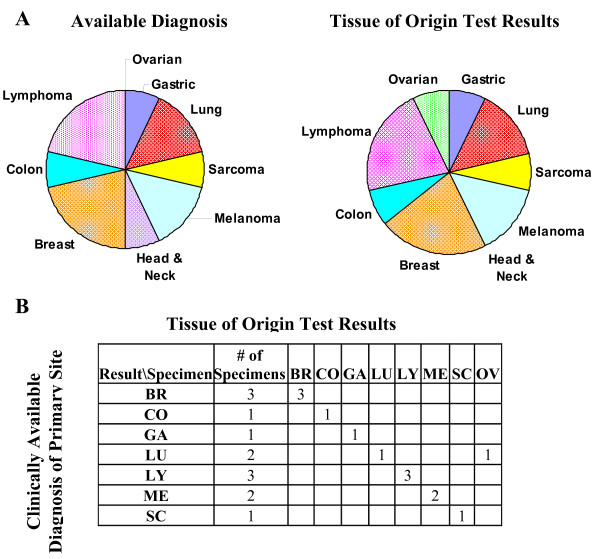
**Distribution of the available diagnosis and the Tissue of Origin Test result by tissue type**. Pie charts (A) and a confusion matrix (B) were used to compare the available diagnosis with the Tissue of Origin Test results. Among the cases that were on-panel, the Tissue of Origin Test had a 92.3% (12/13) agreement with the available diagnosis. The Tissue of Origin Test Result for the off-panel case of squamous cell carcinoma of the head and neck was lung. Abbreviations are BR = Breast, CO = Colon, GA = Gastric, LU = Lung, LY = Lymphoma, ME = Melanoma, SC = Sarcoma, OV = Ovarian.

In addition to the predicted primary (tissue with the highest Similarity Score), each test result ruled out as a primary site (Similarity Scores < 5) an average of 13 of the 15 tissues on the panel. The distribution of the available diagnoses and Tissue of Origin Test results by tissue and a confusion matrix of these data are shown in Figure 2. Available diagnoses for specimens entered into the study represented nine independent tissues (Figure 2A). The Tissue of Origin Test Result for the off-panel case of squamous cell carcinoma of the head and neck was lung.

## Discussion

The Pathwork Tissue of Origin Test showed a high degree of agreement with the clinically available diagnosis, accurately identifying the primary site for 92.3% of the metastases to brain from primary sites on the Tissue of Origin panel and 86.7% overall (including the off-panel head and neck carcinoma). This rate of agreement is quite comparable to the percent agreement of 87.8% obtained during the clinical validation of the Tissue of Origin Test [10] and is superior to the 66% agreement seen in similar blinded studies in which a pathologist uses immunohistochemistry to identify the primary [9]. In addition to providing information about the most likely primary site, each Test also provided information that definitively ruled out an average of 13 tissues as sites of origin.

The cases in this study were not selected for having primary sites on the Tissue of Origin Test panel. Nonetheless, 93% of the specimens' primary sites were available on the Tissue of Origin Test panel. For the off-panel specimen, the Test generated 13 tissue rule-outs. The Tissue of Origin Test results were also consistent with the expected distribution of primary sites for the tumors most commonly metastasizing to brain [1].

Currently, a version of the test has become available that works with formalin-fixed paraffin-embedded (FFPE) specimens [13]. The ability to use FFPE enhances the clinical utility of the Tissue of Origin Test since FFPE is the more commonly available clinical material. While the method of processing the total RNA and classification algorithm used are different in the frozen and FFPE versions of the Tissue of Origin Test, they both use microarray expression data and multigene classifiers to distinguish between the same 15 tissue types described in this study. The FFPE Test output, similar to the frozen Test, is 15 similarity scores that range from 0 (very low similarity) to 100 (very high similarity). Importantly, clinical validation studies have shown that performance of the FFPE Test had an accuracy of 88.5% in a set of 462 FFPE specimens which is equivalent to the frozen Test [10,13].

A large number of screened samples were not eligible for entry into the study because they contained extensive necrosis. The validation studies for the Tissue of Origin Test did not include samples with necrosis > 20%. Tumor necrosis in brain metastases is often extensive, leaving recognizable tumor tissue only at the periphery of the lesion and around blood vessels. Tissue macrodissection to remove necrotic tissue performed by a trained pathologist might be used successfully with the Tissue of Origin Test for highly necrotic samples. The FFPE version of the Tissue of Origin Test can be used with specimens containing up to 40% necrosis (minimum 60% tumor tissue) making it more useful in clinical practice for brain tumors. Additionally, tumor punches of FFPE tissue blocks can be performed to select non-necrotic tumor tissue for the Tissue of Origin Test.

Our study shows that the Tissue of Origin Test is a useful diagnostic tool for brain metastases and can serve as a tool for neuropathologists as they classify metastatic brain cancers. It is expected that improved accuracy in the diagnosis of the primary sites for metastatic brain cancer will aid in selecting further diagnostic tests and optimal therapy. Not only would it guide the clinical workup to locate the primary tumor but it would allow oncologists to treat these patients with more specific targeted therapies. Several cancers that commonly metastasize to the brain such as breast, ovarian and small cell lung carcinomas are highly responsive to current therapies and an accurate diagnosis would be of considerable benefit to these patients. Accurate identification of the primary site could eventually lead to improved clinical outcomes. It has been shown that identifying colon as the primary site for carcinoma of unknown primary (CUP) patients results in improved clinical outcome [14,15]. These CUP patients had better response to colon-cancer specific treatment regimens than they did to empiric CUP therapy with paclitaxel and carboplatin-based regimens.

## Conclusions

The Pathwork Tissue of Origin Test has high accuracy in identifying the primary site for brain metastases cases. This test could be a valuable tool for pathologists as they classify metastatic brain cancers potentially resulting in improved clinical management of these patients.

## Competing interests

Three of the authors are employees of Pathwork Diagnostics, manufacturer of the Tissue of Origin Test.

## Authors' contributions

AHBW, WDH, and RP conceived and designed the study, and reviewed the manuscript. JCD assisted in the performance of the study, interpreting the data, and reviewed the manuscript. HW assisted in the performance of the study. SRV was the custodian of the UCSF Neurological Surgery Tissue Bank, reviewed the pathology of the samples, and provided clinical expertise on these cases. AL participated in interpreting the data and authored the manuscript. All authors have read and approved of the final manuscript.

## Supplementary Material

Additional file 1 **Tissue of Origin Test Results with all 15 Similarity Scores for the Brain Metastases Cases**. This table contains complete Tissue of Origin Test results, including all 15 similarity scores, for the 14 brain metastases cases in this study. The highest similarity score for each case is highlighted and represents the identified Tissue of Origin for that case. Click here for file
