# Supplementary material for: Gene expression profiles help identify the Tissue of Origin for metastatic brain cancers
Source: Diagn Pathol. 2010 Apr 26;5:26. doi: 10.1186/1746-1596-5-26 (PMC2867958; doi:10.1186/1746-1596-5-26)
Supplement: Additional file 1 — Tissue of Origin Test Results with all 15 Similarity Scores for the Brain Metastases Cases. This table contains complete Tissue of Origin Test results, including all 15 similarity scores, for the 14 brain metastases cases in this study. The highest similarity score for each case is highlighted and represents the identified Tissue of Origin for that case. [file 1746-1596-5-26-S1.DOC]

| **Additional file 1: Tissue of Origin Test Results with all 15 Similarity Scores for the Brain Metastases Cases** | | | | | | | | | | | | | | | | | |
| --- | --- | --- | --- | --- | --- | --- | --- | --- | --- | --- | --- | --- | --- | --- | --- | --- | --- |
| **Case ID** | **Available Diagnosis** | **Tissue of Origin Test Result** | **Similarity Scores for the 15 tissue types on the Tissue of Origin Test panel*** | | | | | | | | | | | | | | |
|  |  |  | **BL** | **BR** | **CO** | **GA** | **GC** | **KI** | **LI** | **LU** | **LY** | **ME** | **OV** | **PA** | **PR** | **SC** | **TH** |
| E | BR | BR | 0.2 | 90.6 | 1.5 | 0.1 | 0 | 0.1 | 0.1 | 5.8 | 0 | 0 | 1.1 | 0.3 | 0.1 | 0 | 0.1 |
| F | BR | BR | 0.2 | 81 | 2.5 | 0.1 | 0 | 0.5 | 0.2 | 6.3 | 0 | 0.1 | 8.9 | 0.1 | 0.1 | 0.1 | 0.1 |
| K | BR | BR | 0.7 | 90.5 | 1.3 | 0.1 | 0.3 | 0.8 | 0.4 | 2.8 | 0 | 0.2 | 1.9 | 0.3 | 0.1 | 0.4 | 0.3 |
| H | LY | LY | 0.3 | 0.5 | 0.3 | 1.3 | 0.4 | 0.6 | 0.8 | 1.1 | 90.4 | 1 | 1.5 | 0.2 | 0.1 | 1.4 | 0.1 |
| I | LY | LY | 0.2 | 0.9 | 0.4 | 1.9 | 0.7 | 1 | 1.2 | 1.1 | 87.7 | 0.9 | 1.3 | 0.6 | 0.1 | 1.8 | 0.2 |
| O | LY | LY | 0.1 | 0.2 | 0.1 | 0.5 | 0.3 | 0.6 | 2 | 0.3 | 94.4 | 0.2 | 0.7 | 0.1 | 0 | 0.4 | 0.1 |
| G | LU | LU | 0.2 | 3.3 | 2.3 | 1 | 0.9 | 2.4 | 1.6 | 62.6 | 0.7 | 0.3 | 18.9 | 1.2 | 0.8 | 0.7 | 3.2 |
| N | LU | OV | 0.1 | 0.3 | 0.4 | 0.1 | 0.1 | 0.3 | 0.3 | 8.3 | 0.1 | 0 | 89.4 | 0.2 | 0.1 | 0.1 | 0.2 |
| C | ME | ME | 0.7 | 1.1 | 0.5 | 1.5 | 1 | 1.9 | 1.4 | 7.2 | 0.4 | 77 | 3 | 0.7 | 0.3 | 2.8 | 0.4 |
| D | ME | ME | 0.6 | 2.6 | 0.6 | 1.1 | 1.4 | 1.9 | 2.1 | 4.5 | 1.2 | 75.9 | 3.3 | 0.5 | 0.3 | 3.2 | 0.7 |
| J | CO | CO | 0.5 | 2.2 | 82.4 | 1 | 0.5 | 0.7 | 0.7 | 3.8 | 0.7 | 0.3 | 2.8 | 2.9 | 0.2 | 1.1 | 0.4 |
| M | GA | GA | 2.4 | 4.7 | 5.4 | 35 | 1.7 | 2.7 | 3.5 | 21.5 | 0.8 | 0.3 | 10.8 | 6.6 | 0.5 | 3.3 | 0.8 |
| A | SC | SC | 2.4 | 2.6 | 0.7 | 0.9 | 4.5 | 4.6 | 2.7 | 1.4 | 0.9 | 2.5 | 8.4 | 0.6 | 0.4 | 65.2 | 2.2 |
| L | HN | LU | 0.2 | 8.4 | 3.2 | 0.4 | 0.8 | 1.2 | 0.7 | 77.3 | 0.4 | 0.6 | 2.9 | 0.5 | 0.2 | 2.5 | 0.7 |
| * The Tissue of Origin Test result is based on the highest similarity score. The highest similarity score for each case is highlighted.  Abbreviations Used: BL=Bladder; BR=Breast; CO=Colon; GA=Gastric; GC=Testicular Germ Cell; KI=Kidney; LI=Liver; LU=Lung; LY=Lymphoma; ME=Melanoma; OV=Ovarian; PA=Pancreas; PR=Prostate; SC=Sarcoma; TH=Thyroid | | | | | | | | | | | | | | | | | |
